# Clinical, logistic, and geographic factors in ensuring adequate access to implant removals: A cross-sectional survey of public facilities and GIS modeling of geographic access in two districts of Senegal

**DOI:** 10.3389/fgwh.2022.899543

**Published:** 2022-10-25

**Authors:** Aurélie Brunie, Caleb Parker, Salif Ndiaye, Fatou Ndiaté Rachel Sarr Aw, Emily B. Keyes, Elena Lebetkin, Etienne Dioh, James MacCarthy, Marème Mady Dia Ndiaye

**Affiliations:** ^1^ Global Health and Population Research Department, FHI 360, Washington, DC, United States; ^2^ Data and Analytics Department, FHI 360, Durham, NC, United States; ^3^ Centre de Recherche pour le Développement Humain (CRDH), Dakar, Senegal; ^4^ IntraHealth International, Dakar, Senegal; ^5^ Community and Health Systems Department, FHI 360, Durham, NC, United States; ^6^ Product Development and Introduction Department, FHI 360, Durham, NC, United States; ^7^ Global Forest Watch Team, World Resources Institute, Washington, DC, United States; ^8^ Sénégal Ministère de la Santé et de l’Action Sociale, Direction de la Sante de la Mère et de l’Enfant, division Planification Familiale, Dakar, Senegal

**Keywords:** Senegal, sub-Sahara Africa (SSA), contraception, contraceptive implant, long-acting reversible contraception (LARC), removals, referrals, readiness

## Abstract

**Introduction:**

Ensuring adequate access to contraceptive implant removal services requires an understanding of potential clinical, logistical, and geographic challenges.

**Methods:**

We conducted a cross-sectional survey of 39 public health facilities in two districts of Senegal. To assess facility readiness, we reported the proportion of facilities meeting all minimum conditions for regular and difficult implant removals. We then describe characteristics of referral networks. Geographic access modeling was conducted in a geographic information system to estimate the proportion of women of reproductive age living within specific travel times of facilities ready for regular and difficult removals.

**Results:**

72% of facilities met all conditions for regular removals, and 8% for difficult removals. In both cases, the main gaps related to equipment availability (79% of facilities had the minimum equipment for regular removals and 8% for difficult removals). 72% of facilities organized in three referral networks sent clients to other facilities for cases they could not manage. Of 11 receiving or single-network facilities, seven were ready for regular removals and one for difficult removals. Altogether, 36% of women in Dakar Centre and 99% of women in Kolda lived within two hours of a facility that was equipped to handle regular removals, compared to 15% and 69%, respectively, for difficult removals.

**Conclusion:**

Data such as those provided in this assessment are important to provide a realistic picture of the state of readiness of the health system and its ability to meet the inevitable demand for implant removals. Referral networks should be considered as an emerging strategy to avail sufficient capacity at the systems level, including for managing difficult removals. However, careful thought should be given to the location of facilities that are ready to receive cases in order to target upgrades.

## Introduction

Family planning use in West Africa has historically been among the lowest in the world, but tremendous progress has been made since the establishment of the Ouagadougou partnership in 2011. Availability and use of contraceptive implants in sub-Saharan Africa have grown substantially over the past decade, including in West Africa (1). Among the nine Francophone countries forming the Ouagadougou partnership, implants are now the most used method by women who are married or in union in three countries (2). In Senegal, modern contraceptive prevalence among married women has risen from 14.3% in 2012 to 28.5% in 2019 (3). Implants are the second most used method after injectables; implant prevalence in the method mix has grown from 9% to 31% between 2011 and 2017, with 96% of implants being inserted through public sector facilities in 2017 (4, 5).

Currently available subdermal contraceptive implants offer a duration of protection against pregnancy ranging from three to five years, though users may discontinue them early at any time of their choosing. Like insertion, implant removal requires a surgical procedure by a qualified provider, as well as the availability of the necessary commodities. In addition, while most removal procedures should be simple, minor, and quick with appropriate training and equipment (referred to as “regular removals” throughout this paper), they may occasionally be difficult procedures, including in cases where the implant is deeply embedded in the arm or not palpable. These cases require a higher level of care to have the implant removed (referred to as “difficult removals” throughout this paper) (6).

While there is limited evidence regarding women's access to health facilities for implant removal in low- and middle-income countries (LMIC), available data suggest there may be a gap between the capacity for insertion and for removal, with more providers offering insertion than removal services, as well as reports of failed removal attempts (7–10). Possible reasons for this include delayed demand for removals compared to insertion, greater technical difficulty of procedure, need for autonomy in making decisions related to how to perform the procedure compared to insertion, limited opportunities to maintain skills due to low client load, and limited availability of d sterile surgical instruments and disposable commodities (8, 9). It may not be feasible to establish removal services at all levels of the health system where insertion is available, therefore increasing focus on referral systems can be an important strategy to manage these variables and to ensure universal, reliable, rights-based access to removal (8). Appropriate referral networks are also an emerging strategy for management of difficult removals that require specialists and imaging equipment in high- and upper-middle income countries (11–14).

The Global Implant Removal Task Force, started in late 2015, has contributed recommendations and resources to bring awareness to the issue of implant removal and improve readiness to offer quality removal services (8, 15). One immediate challenge program managers in LMIC face, however, is to get a solid understanding of the current state of readiness of their system to manage removals. This includes an understanding of potential clinical challenges and logistical health system issues, as well as possible geographic challenges for clients in reaching a facility with appropriate removal services, especially as referral facilities may be farther away than insertion facilities and thus less accessible to clients.

The analyses presented in this paper were intended to provide such evidence to inform potential strengthening of removal services in Senegal. This is, to our knowledge, the first undertaking of this kind in LMIC documented in the peer-reviewed literature. First, we aimed to assess service readiness for regular and difficult removals in public sector facilities in two districts. Second, we examined existing referral networks with the goal of assessing whether they were rational in the context of removal services, i.e., if clients are being referred to facilities that are ready to manage removal cases. Third, we used a geographic information system (GIS) to model travel time to services and generate estimates of client access.

## Methods

### Study design and data collection

We conducted a cross-sectional survey of all public health facilities offering insertion of long-acting reversible contraceptives (LARCs) in two districts of Senegal - urban Dakar Centre and primarily rural Kolda). This study was part of a larger project that also examined client and provider experiences with removal services; these results are presented elsewhere (16, 17). Trained research assistants administered the health facility questionnaire to the in-charge of the facility or designee (maternal and child health department in-charge or midwife); after they provided written consent for the health facility, these respondents could solicit input from other staff based on expertise relevant to specific questions.

Research assistants collected data in-person between 9 December 2019 and 29 May 2020 using a structured tablet-based questionnaire programed in Open Data Kit to minimize data entry error. Topics included availability of trained staff, schedule for removal services, equipment and consumables (including direct observation to confirm availability on the day of the visit), and referral mechanisms. Tablets recorded geocoordinates for each facility. At the end of each day of data collection, data were transferred from the tablets to a secure server using a cellular network or wireless connection. To ensure participant confidentiality, data were deleted from the tablets after transfer. Data quality checks were performed in the field by data collector supervisors and an analyst at FHI 360 in North Carolina conducted additional quality checks.

### Facility readiness

We performed descriptive analyses of survey data in Stata 13 and geospatial analyses in ArcGIS Pro. In assessing facility readiness for regular and difficult removals, we considered three components: general service readiness, human resources, and observed availability of minimum equipment and consumables. Our approach was adapted from measures of readiness, or facility capacity, as used by the World Health Organization's Service Availability and Readiness Assessments (SARA) (18, 19). Minimum requirements for each component were established in discussion with study partners in Senegal and a clinical expert in the United States (Table 1). X-ray was not included in the equipment component of the readiness assessment because all available implants (Jadelle, Nexplanon, Implanon) are visible with ultrasound equipment, while Jadelle is only visible on an x-ray. Furthermore, no facilities had both an x-ray machine and staff trained to use it. When facilities were not able to remove implants on-site, we assigned facilities to referral networks based on their survey responses. Using this framework, we assigned each facility a readiness level (not ready, regular removal, difficult removal) and referral category (primary, referral, or single-network).

**Table 1:** Components of readiness assessment to handle regular and difficult removals.

Resources required for:	Regular removals	Difficult removals
***General service readiness***		
Availability of equipment for infection prevention - Running water, decontamination buckets, safety buckets, and soap	Yes	Yes
Facility offering implant removals at least five days per week	Yes	Yes
***Human resources***		
At least one staff person trained in implant removal	Yes	Yes
At least one staff person trained in use of ultrasound	No	Yes
***Observed availability of minimum equipment***		
Syringes, local anesthetic, sterile band aids, scalpel with blade, curved and straight forceps, antiseptic, and cotton balls or sterile gauze	Yes	Yes
Modified vasectomy forceps, ultrasound, sterile towels, examination table, sterile surgical drape, sterile equipment tray	No	Yes

### Geospatial analysis

The access modeling and other geospatial analyses were conducted in ArcGIS Pro and assumed clients traveled to the nearest facility ready to handle regular or difficult removals, as per the facility readiness analysis. Due to substantial differences between urban and rural transportation networks, we modelled access to services using two different approaches.

For densely populated urban district of Dakar Centre, where traffic jams heavily impact travel time and most clients travel to health facilities by public transportation, we based the model on the road network. We used geocoordinates of the center point of the 425 neighborhoods that were part of the intended catchment areas of the facilities in the assessment, and modeled travel time from each neighborhood center point to the nearest health facility. Travel time assumptions include a 50-min wait time for public transportation pick up based on conversations with the site team, and a 10 kph travel speed–the average speed of public transportation for this area, which was estimated by using Google maps navigation between random points in the study area at 8 am and 5 pm on weekdays. For each neighborhood center point, the neighborhood boundary was estimated using Theissen polygons, which defines an area around a point where every location is nearer to this point than to all others. Travel time results for each neighborhood center point were applied to the entire neighborhood polygon.

In the larger, more rural Kolda District, where people live far from roads and public transport is not readily available, we included both roads and surfaces without roads. in the model. This model assumed women walk across surfaces without roads and then take motorized transport once they reach a roadway. The travel speeds differed by road type and ranged in speeds from 10 to 60 kph, while the non-road surfaces were given a general walking speed of 1 to 2.5 kph depending on the surface type. The models were run to identify areas by hourly travel segments (e.g., 0–1 h, 1–2 h, 2–3 h) from each health facility, and the total populations living in those travel segment areas were summed.

**Figure 1:**
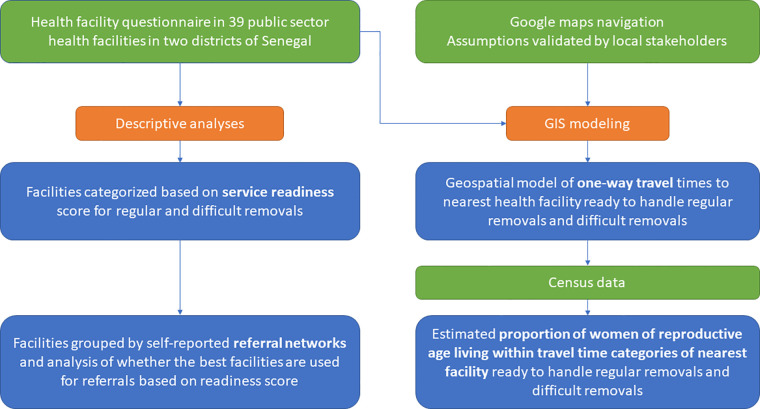
Summary of design and analyses.

For both locations, a modeled population data layer based on census data of women of reproductive age for the year 2018 (20) used as a proxy for women of reproductive age was layered onto the Dakar Centre neighborhoods or resulting travel time area segments in Kolda district and summed by segment to estimate the proportion of women of reproductive age living within each travel time category. Figure 1 describes the study design and analysis approach.

The Comité National d'Ethique pour la Recherche en Santé in Senegal and FHI 360's Protection of Human Subjects Committee in the United States approved the study.

## Results

We collected data from 39 of 42 health facilities offering LARC insertions; data collection in the remaining three facilities was not possible due to the onset of the COVID-19 pandemic and associated restrictions. The majority of the facilities were health posts (82%) and the remaining were health center level I (15%) and health center level II (3%).

### Service readiness

Overall, 72% of facilities met al.l conditions for regular removals, while 8% of facilities were ready for difficult removals. General readiness criteria for regular and difficult removals were fulfilled by 92% of facilities. All facilities had suitable human resources for regular removals (at least one person trained in removal), but only 18% met the additional requirement of having at least one staff person trained to use ultrasound for difficult removals. 79% had the necessary equipment and consumables for regular removals, while 8% were fully equipped to handle difficult removals (Table 2).

**Table 2:** Readiness to handle regular and difficult removals by component.

*Component:*	Dakar Centre (*n* = 13) %	Kolda (*n* = 26) %	Total (*n* = 39) %
**Regular removals**	**77**	**69**	**72**
**General service readiness**	**100**	**88**	**92**
*Running water*	*100*	*88*	*92*
*Decontamination buckets*	*100*	*100*	*100*
*Safety boxes*	*100*	*100*	*100*
*Soap*	*100*	*100*	*100*
*Facility offers implant removals 5 + days per week*	*100*	*100*	*100*
**Human resources: 1 + staff trained in implant removal**	**100**	**100**	**100**
**Minimum equipment**	**77**	**81**	**79**
*Syringes*	*85*	*96*	*92*
*Local anesthetic*	*92*	*92*	*92*
*Sterile band aids*	*85*	*96*	*92*
*Scalpel with blade*	*85*	*92*	*90*
*Curved forceps*	*85*	*100*	*95*
*Straight forceps*	*92*	*96*	*95*
*Sterile gauze, or antiseptic and cotton balls*	*100*	*100*	*100*
**Difficult removals (same as regular removals plus below)**	**15**	**4**	**8**
**General service readiness**	**100**	**88**	**92**
**Human resources: 1 + staff trained to use ultrasound**	**31**	**12**	**18**
**Minimum equipment**	**15**	**4**	**8**
*Modified vasectomy forceps*	*54*	*58*	*56*
*Ultrasound*	*38*	*12*	*21*
*Sterile towels*	*77*	*73*	*74*
*Examination table*	*100*	*100*	*100*
*Sterile dry surgical drape*	*54*	*58*	*56*

A component of general service readiness, infection prevention equipment was universally available except running water, with three facilities in Kolda reporting they did not have access to running water. Availability of consumables for regular removals was confirmed through observation for all items. For equipment, it ranged from 90% for scalpel with blade to 100% for the sterile gauze or antiseptic and cotton balls. In terms of additional resources for difficult removals, the least commonly available items included vasectomy forceps (56%) and a sterile surgical drape (56%). 21% of facilities were equipped with an ultrasound machine, but only 18% had both a machine and staff trained to operate it (Table 2). While not considered in the general readiness or human resources components, it is important to note that only 64% of facilities had functioning autoclaves and only 69% of facilities had at least two providers trained in implant removal (not shown).

### Referral networks for difficult removals

Twenty-eight facilities (72%) indicated referring clients to other facilities for difficult removals they were unable to handle themselves, with receiving facilities including two of the facilities in our sample and one facility for which we were not able to obtain data due to COVID-19 restrictions. The remaining nine facilities (23%) were not connected to other facilities *via* a referral network; they did not refer clients nor did they receive referred clients for removals. The three referral networks comprised 20, 7, and 4 facilities respectively, inclusive of the receiving facility (Table 3).

**Table 3:** Referral networks for difficult removals.

Reference facility	Facilities referring to reference facility
**Dakar**	
Health center #1 ^○^	1 (health center) ^◊^; 5 (3 health post, 2 health center) ^○^
Health center #2 ^○^	–
Health center #3 ^◊^	–
Health post #1 ^□^	–
Health post #2 ^○^	–
Health post #3 ^□^	–
Health post #4 ^□^	–
**Kolda**	
Hospital #1^a^	1 (health post) ^◊^; 2 (health post) ^○^
Health center #1 ^○^	12 (health post) ^○^; 7 (health post) ^□^
Health post #2 ^□^	–
Health post #3 ^○^	–
Health post #4 ^○^	–

Facility ready for removals: ◊ normal and difficult; ○ normal only; □ neither normal nor difficult.

^a^Hospital #1 in Kolda did not complete a facility assessment due to COVID-19 restrictions. However, it is included here and in the GIS modeling as it was reported as a referral facility by several other health facilities.

Two of the three receiving facilities in our sample were equipped to handle regular removals, but neither one met our criteria for difficult removals. The nine facilities that were not part of a referral network included one facility equipped to handle regular and difficult removals, 4 facilities equipped to handle regular removals but not difficult removals, and four facilities that did not meet the criteria for managing either regular or difficult removals.

### Travel time to access removal services

**Figure 2:**
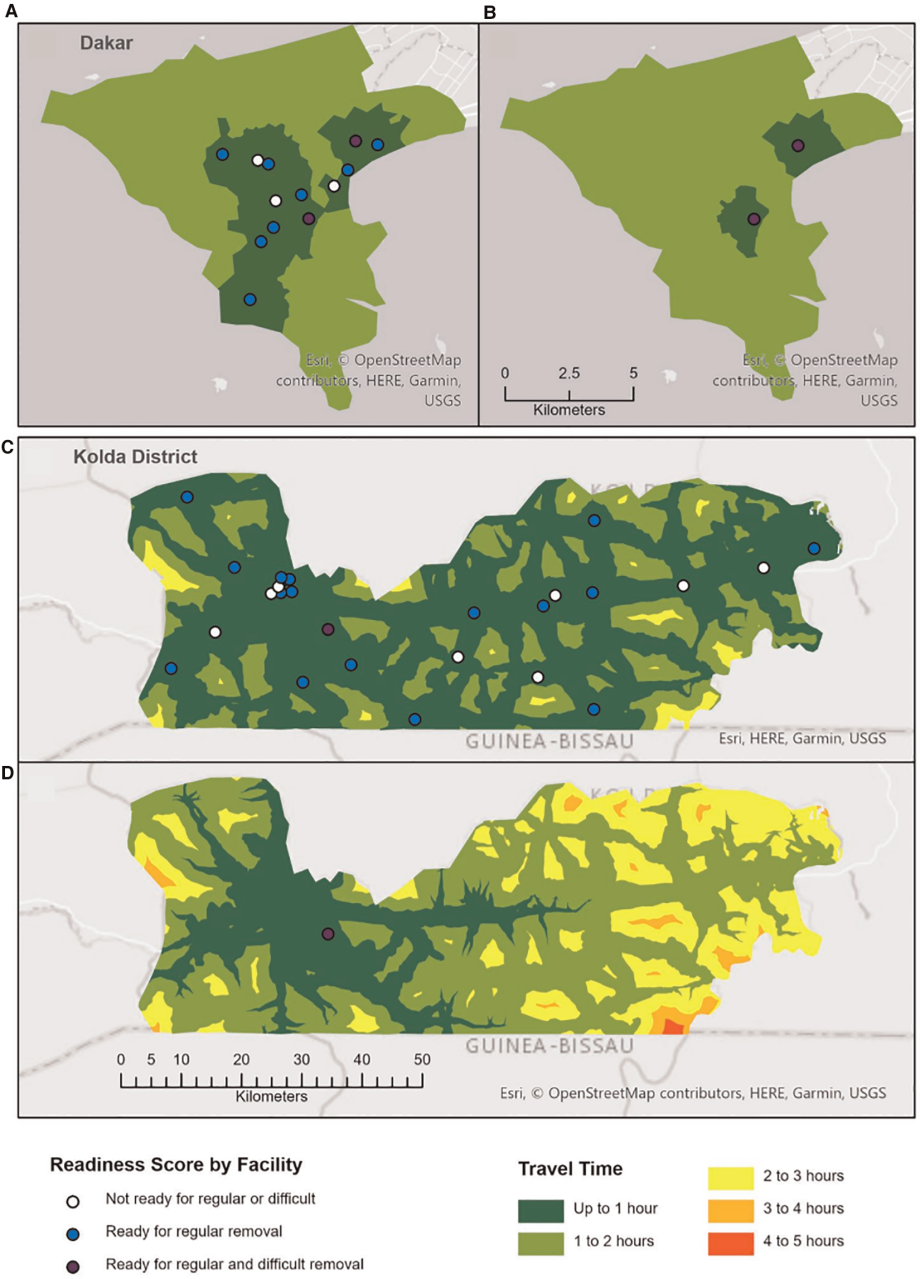
Travel time access to removal services. (**A**) Regular removal in Dakar. (**B**) Difficult removal in Dakar. (**C**) Regular removal in Kolda District. (**D**) Difficult removal in Kolda District.

Figure 2 shows the results of a geospatial model with the estimated one-way travel times that women take to reach the nearest health facility ready to handle regular removals and difficult removals. For example, women whose residence is located in areas colored in dark green live within one hour of the nearest facility that can manage regular/difficult removals. Table 4 shows the proportion of the population of women of reproductive age living within specific travel times of facilities.

**Table 4:** Proportion of the population of women of reproductive age living within specific one-way travel times of facilities.

	Dakar centre facility ready for		Kolda facility ready for	
	Regular removals	Difficult removals	Regular removals	Difficult removals
*Hours of travel time:*	%	%	%	%
0–1	35.5	15.2	98.9	69.4
1–2	64.5	84.8	1.0	28.4
2–3	0.0	0.0	0.1	2.1
3+	0.0	0.0	0.0	0.0
Total	100.0	100.0	100.0	100.0

Results show the proportion of women of reproductive age. It is assumed that the same general distribution is expected to apply to implant users.

Under our assumptions, 36% of women in Dakar Centre live within one hour of a facility ready to handle regular removals and only 15% live within an hour of a facility for difficult removals. All women in Dakar Center live within two hours of regular and difficult removal though most women in Dakar Centre require between one and two hours total (including wait time for transportation) to reach a facility that can manage regular (65%) or difficult (85%) removals. For Kolda, 99% of women live within one hour of a facility ready to handle regular removals. For difficult removals, 69% of women in Kolda live within one hour of a facility ready to manage these cases, 28% within two hours, and 2% at more than two hours.

## Discussion

Our findings from public sector facilities in two districts of Senegal present rare evidence on the state of readiness of implant removal services in LMICs through a comprehensive examination of clinical, logistic, and geographic factors. Our findings highlight some successes, as well as areas to strengthen management of both regular and difficult removals.

We found that almost three quarters of facilities were ready to manage regular removals. All facilities had staff trained on both insertion and removal. We observed some minor gaps related to the availability of equipment and consumables for regular removals which aligns with findings from the provider interviews from this study reported elsewhere (16). Importantly, this study was performed in public facilities benefiting from NGO support, therefore as found in other studies, the availability of equipment and consumables may be high compared to other facilities (7, 21–23). Assessments in Burkina Faso, Democratic Republic of the Congo, Nigeria, and Tanzania noted some similar challenges, notably related to the availability of trained providers to perform removals, especially difficult removals, and the availability of equipment and consumables (23–26).

Upon presenting result to stakeholders in Senegal, the recommendation arose to measure stock levels in other areas of the country and to ensure equipment and consumables were included in routine quantification exercises. The ability to sterilize equipment, normally assessed through the presence of autoclaves, was ultimately not included in the assessment because prevalence was low and it was reasoned that other strategies were available for sterilizing equipment; however, this assumption cannot be verified because information on alternative strategies was not collected as part of the survey. Programmatic assessments should examine whether appropriate strategies are in place.

Although global guidance for difficult implant removal is available (27) and there is some evidence of the feasibility of these protocols in African settings, little is known on the broader availability of these services, including referral mechanisms (28). In contrast to regular removals, few facilities were ready to manage difficult removals. While a smaller number of referral centers may be sufficient to manage difficult cases since these are likely to be rare, referral mechanisms should be in place to ensure that clients receive appropriate care and to support voluntary discontinuation. Our analyses found that current referral networks were not rational for the purposes of effectively managing difficult removal cases, as according to our analysis, the receiving facilities were not capable of handling difficult removals. In addition, we found that some facilities that were not ready to handle difficult (and possibly also regular) removals did not appear to be referring clients.

In modeling one-way travel times to the nearest facility meeting criteria for managing removals, we found that, for both regular and difficult removals, the majority of women in Dakar Centre lived within one to two hours of an appropriate facility, while most women in Kolda had a travel time of one hour or less. Importantly, these estimates must be doubled to include travel time back to women's homes and also do not account for additional time spent at the facility waiting for and receiving services. Separate data collected from clients show these time scan range from 81 to 90 min (results not shown). When interpreting findings, managers should consider that these results are based on the nearest facility equipped to manage referral cases. In practice, women are likely to return first to the clinic in which their implant was inserted, as was also found in another study in Ghana (7). Given that this clinic may not be equipped to manage removals and that referral pathways are misaligned with readiness to manage removals, these results are likely to underestimate the actual time requirements for women as they seek to obtain a removal. By utilizing modeling techniques similar to those applied to examine access to other services such as emergency obstetric and newborn care (29), our study also contributes additional evidence demonstrating the value of using geospatial data to improve services.

Taken together, findings on referral networks and travel times indicate that careful thought needs to be given to referral mechanisms for difficult removals, as well as where facilities are not ready to manage regular removals. This includes recommendations on deciding whether removals should be attempted on site or referred for advanced care. This also should go hand in hand with strengthening receiving facilities to ensure that care of clients presenting to clinics upon being referred is reliable and successful. Creative strategies combining facility upgrades and potential alteration of the current configuration of referral networks should be considered to optimize access to removal services. To understand what optimizing access means for clients, it is important to investigate if clients are willing to travel to key facilities that could serve as referring facility.

This study was intended to synthesize information on the state of implant removal services and to visualize referral networks and access to removal services with a view to inform strategic thinking by the Ministry of Health and implementing partners. Our analyses are limited to public facilities in two districts of Senegal and may not adequately represent other areas of the country. Survey questions on availability of trained staff did not differentiate between training on regular and difficult removals. Analyses of travel times are based on a combination of study data and assumptions, and the modeling was conducted differently for these two sites to account for the context of urban vs. rural transportation. Although these assumptions are anchored in discussions with local experts familiar with local conditions, they do not allow for heterogeneity in transport patterns or variations in wait times or delays due to traffic jams. The modeling in Dakar that considers a 50-minute wait time for transport may exaggerate travel time for women living within walking distance to the facility, for example. Findings should be interpreted with these limitations in mind and taken to represent an order of magnitude rather than an exact value. Calculations on the proportion of the population living within specific travel times of facilities are based on available estimates on the distribution of women of reproductive age that may not be proportionate to the distribution of implant users and cases of regular and difficult removals.

## Conclusion

Ensuring that capacity for removal keeps pace with the inevitable demand that will flow from the unprecedented growth in implant use is an essential aspect of rights-based family planning and informed choice. Supporting program managers with data such as the ones provided in this assessment is important to provide a realistic picture of the state of readiness of the health system to manage removals and identify potential areas for strengthening. Referral networks should be considered as an emerging strategy to avail sufficient capacity at the systems level, including for managing difficult removals. Using GIS can be a useful approach to assess the potential geographic constraints that clients may face in accessing services.

## Data Availability

The datasets presented in this study can be found in online repositories. The names of the repository/repositories and accession number(s) can be found below: Underlying data and documentation are available in the Harvard Dataverse at https://doi.org/10.7910/DVN/DLW6F8.
